# Living-Donor Liver Transplantation and Hepatitis C

**DOI:** 10.1155/2013/985972

**Published:** 2013-01-21

**Authors:** Nobuhisa Akamatsu, Yasuhiko Sugawara

**Affiliations:** ^1^Department of Hepato-Biliary-Pancreatic Surgery, Saitama Medical Center, Saitama Medical University, 1981 Tsujido-cho, Kamoda, Kawagoe, Saitama 350-8550, Japan; ^2^Artificial Organ and Transplantation Division, Department of Surgery, Graduate School of Medicine, University of Tokyo, 7-3-1 Hongo, Bunkyo-ku, Tokyo 113-8655, Japan

## Abstract

Hepatitis-C-virus- (HCV-) related end-stage cirrhosis is the primary indication for liver transplantation in many countries. Unfortunately, however, HCV is not eliminated by transplantation and graft reinfection is universal, resulting in fibrosis, cirrhosis, and finally graft decompression. In areas with low deceased-donor organ availability like Japan, living-donor liver transplantation (LDLT) is similarly indicated for HCV cirrhosis as deceased-donor liver transplantation (DDLT) in Western countries and accepted as an established treatment for HCV-cirrhosis, and the results are equivalent to those of DDLT. To prevent graft failure due to recurrent hepatitis C, antiviral treatment with pegylated-interferon and ribavirin is currently considered the most promising regimen with a sustained viral response rate of around 30% to 35%, although the survival benefit of this regimen remains to be investigated. In contrast to DDLT, many Japanese LDLT centers have reported modified treatment regimens as best efforts to secure first graft, such as aggressive preemptive antiviral treatment, escalation of dosages, and elongation of treatment duration.

## 1. Introduction

Since the first successful application of living donor liver transplantation (LDLT) in 1990 [1] and subsequent successful LDLT for adult recipient in 1994 [2], the use of live donors for liver transplantation has been widely applied to adult recipients where the availability of deceased-donors is severely restricted, like in Japan [3], and also accepted as a solution to the cadaveric donor shortage in Western countries [4].

End-stage liver disease caused by chronic hepatitis C virus (HCV) infection is the leading cause of liver transplantation in developed countries [5, 6], including Japan [7]. Unfortunately, liver transplantation does not cure HCV-infected recipients, but re-infection of HCV universally occurs and disease progression is accelerated compared with that in the nontransplant population, resulting in poor outcomes for HCV-infected recipients [8].

The aim of this paper was to overview the current trends and controversies in LDLT for patients with HCV in relation to the perspectives from deceased-donor liver transplantation (DDLT).

## 2. Natural History of Hepatitis C after Orthotopic Liver Transplantation

Accumulating perspectives of disease recurrence in HCV-infected recipients have been obtained in DDLT within the last two decades. HCV reinfection occurs just after reperfusion followed by a rapid increase in HCV ribonucleic acid (RNA) levels within 4 postoperative months [9]. The histologic features of liver injury usually resemble those of nontransplant HCV hepatitis typically developing after 3 months, but the clinical presentation, severity, and outcome are extremely heterogeneous and more profound compared to those in immune competent patients [10]. Progression to cirrhosis usually takes 9 to 12 years after liver transplantation with a linear progression of histologic fibrosis [10, 11]. A less common, but well-documented, form of recurrence is called fibrosing cholestatic hepatitis (<10%), possibly mediated by a direct cytopathic mechanism under an extremely high viral load and immune-compromised condition. Graft failure occurs in 50% of recipients within a few months after fibrosing cholestatic hepatitis develops [12]. Some HCV-reinfected recipients, however, show no apparent disease progression for at least the first decade and their graft injury remains mild or even absent despite a high viral burden.

Overall, cirrhosis develops in approximately 25% of liver transplant recipients (range 8% −44%) after 5 to 10 years and this percentage is likely to increase with an increase in the follow-up period [10, 11]. Once cirrhosis is complete, survival time is severely decreased and decompression is encountered with cumulative rates at 1 and 3 years of 40% and 60%, respectively, which finally results in graft failure [11, 13].

The development of decompensated cirrhosis due to recurrent hepatitis C is now the most frequent cause of graft failure, patient death, and the need for retransplantation in HCV-infected recipients [11, 13–17]. As a result, survival is significantly decreased compared with other indications, an overall 10% difference at 3 years [18]. In the most recent United Network for Organ Sharing/Organ Procurement and Transplantation Network (UNOS/OPTN) study from the United States, 3-year survival is 78% among 7459 HCV-positive recipients compared with 82% among 20734 HCV-negative recipients (*P* < 0.0001; http://www.unos.org) [19]. 

The poor outcome of HCV-positive recipients has resulted in the divergence in transplant outcomes between HCV-positive recipients and HCV-negative recipients. Improvements in organ preservation, surgical techniques, and postoperative care have dramatically improved the survival of HCV-negative recipients over the last two decades, whereas this has not been the case in HCV-positive recipients for whom outcome has remained unchanged or even worsened over time [19–22].

## 3. Current Status of LDLT

In areas with low deceased-donor organ availability like Japan, the indication of LDLT for HCV cirrhosis is similar to that of DDLT [7], whereas in Western countries, LDLT is conducted in an attempt to alleviate the shortage of donor organs and decrease the mortality among patients awaiting transplants, accounting for only 3% to 4% of all liver transplants [23].

According to the Japan Liver Transplantation Society [24], a total of 6097 LDLTs, comprising 98% of all liver transplants, have been performed till the end of 2010 in Japan. Among those, 3796 were adult cases including 1200 (32%) cases of HCV-related disease as a leading indication for adult LDLT. The 1, 3, 5, and 10 year survival rates of all adult LDLT and those of HCV-positive adults were 81%, 75%, 72%, and 66%, and 78%, 72%, 68%, and 59%, respectively, without difference.

 In the United States, nearly 3000 LDLTs have been performed by the end of 2009, with decreased number of cases annually, comprising only 4.5% of all liver transplants [23, 25, 26].

## 4. LDLT as a Risk Factor for Recurrent Hepatitis C Studies Comparing Outcomes of LDLT and DDLT 

Based on the significant negative impact of recurrent hepatitis C on recipients' outcome, it is critical to identify the factors related to severe recurrent hepatitis C [8, 13]. In the transplant setting, many factors contribute to disease progression compared with nontransplant patients [13], including, viral-related factors [10, 27–36], donor age [17, 37–43], recipient-related factors [32, 44–49], graft and surgical factors [40, 50–57], and immunosuppressive agents [58–75] (Table 1) however, many aspects remain unclear and require further investigation [8]. Among those, the possibility of increased severity of recurrent HCV in LDLT patients had been one of the hottest debates. The benefit of LDLT might be offset if the outcome of LDLT for HCV-positive recipients is worse than that of DDLT.

Early studies raised some negative concerns regarding the outcomes of LDLT in HCV patients, such as a poorer graft outcome and earlier and more aggressive HCV recurrence after LDLT compared with DDLT [144–146]. Several theories have been proposed to explain the differences in HCV recurrence between LDLT and DDLT recipients. One possible explanation is that the intense hepatocyte proliferation that occurs in partial liver grafts may lead to increased viral translation and replication [145, 147–149]. Genetic donor-recipient similarity is another proposed mechanism for more severe HCV recurrence [150, 151]. Recent studies, however, comparing outcomes of LDLT and DDLT in HCV-infected patients have not only failed to identify LDLT as a risk factor for more intense viral recurrence with impaired outcome, but also revealed improved results in LDLT recipients [39, 84–95], which do not support the aforementioned speculations. Alternatively, recent studies favored the theory that outcomes of LDLT for HCV cirrhosis could be better than those of DDLT due to the younger donor age and shorter ischemic time of LDLT grafts. The studies comparing outcomes between LDLT and DDLT in HCV-infected recipients are summarized in Table 2.

While several earlier studies demonstrated impaired patient/graft survival and severe histologic findings in LDLT [144–146], the majority of studies reported equal or even improved outcomes both in patient/graft survival and in fibrosis progression in LDLT [39, 84–95]. Since the large UNOS database study [87] demonstrated comparable short-term (24 months) survival between LDLT and DDLT, subsequent studies with considerable follow-up period have been published demonstrating comparable or even superior outcome in LDLT. Five-year patient survival ranged 71% to 84% in HCV-positive LDLT recipients among studies with sufficient follow-up period [39, 86, 94, 95]. Additionally, as Terrault et al. [92] reported, the learning curve for the LDLT procedure may have a considerable impact on the outcome of LDLT for HCV cirrhosis, which has been repeatedly pointed out by recent authors. Actually, none of reports after 2005 has found impaired outcome in LDLT.

These data should be interpreted with caution, however, because of the important clinical distinction between LDLT and DDLT. At the time of transplantation, DDLT recipients are far sicker than LDLT recipients as represented by a significantly higher MELD score, donor age is higher, and graft ischemic time is longer. Indeed, significantly poorer preoperative condition and older donor age in DDLT recipients were indicated in 7 and 6 studies, respectively, among 16 studies listed in Table 2. Additionally, cold ischemia time is significantly longer in DDLT than that in LDLT in all studies. All these factors, as presented in Table 1, are considered independent prognostic factors for severe HCV recurrence and impaired patient/graft outcome. Actually, Jain et al. [95], who recently reported that both patient/graft survival and histologic findings are better in LDLT, found in a subanalysis of the study that adjusting for MELD score (<25) and donor age (<50) resulted in similar outcomes.

Based on accumulating reports demonstrating comparable outcome of LDLT and DDLT for HCV cirrhosis, and refinement of surgical techniques and management in LDLT, hepatitis C recurrence by itself does not seem to explain the differences in patient/graft survival between LDLT and DDLT, and even improved outcomes could be achieved in LDLT due to the better quality of the graft, younger donors, and less sick recipient condition at the time of transplantation. Furthermore, based on these benefits of LDLT, donor selection to improve outcome of LDLT for HCV positive recipients could be assumed. Selecting younger donors [17] or donors with favorable IL-28B genotype [108, 109] could be possible future issues; however, with the severe lack of live donors, it seems impractical in clinical setting at present. Anyway, LDLT could be strongly recommended for HCV-positive patients whenever it is available.

## 5. Antiviral Treatment 

Antiviral treatments for recurrent hepatitis C after liver transplantation include eradication of the HCV virus before transplantation with the use of pretransplant antiviral treatment, eradication of HCV virus early after transplantation preemptively to prevent graft damage, and treatment for established recurrent hepatitis C in the acute, or more commonly, chronic phase. Regardless of the antiviral treatment timing, interferon (INF), especially pegylated-INF (PEG-INF), in conjunction with ribavirin (RBV), is currently accepted as a standard key drug in achieving high sustained viral response (SVR) rate according to the perspectives obtained in nontransplant populations.

Former two strategies, however, have almost been abandoned in Western countries. Pretransplant treatment is severely limited by poor liver function, a high prevalence of nonresponders, severe cytopenia, and complications, including life-threatening infections [152], and to date, only six studies [153–158] have been published in this phase with differences in the treatment duration (6–14 months versus 2-3 months) and in regimens used (INF only, INF/RBV, or PEG-INF/RBV). Regardless of the approach used, the results are similar, resulting in the prevention of HCV re-infection in about 20% of treated patients with high discontinuation rate and high dose reduction rate [152]. Considering the less severe disease of LDLT recipients as discussed earlier, pretransplant antiviral treatment seems more preferable for LDLT recipients to improve outcome; however, no such trial has been published so far in LDLT setting. This issue also seems remain to be investigated in future studies as with the case in live donor selection issues.

Prophylactic or preemptive antiviral treatment generally means antiviral treatment with INF/PEG-INF and RBV started early posttransplant, without requiring evidence of recurrent hepatitis C. In published studies [159–164] of preemptive antiviral therapy, SVR rates are reported to range from 8% to 34% (5% to 43% for genotype 1 and 14% to 100% for genotypes 2 or 3), with the rates of dose reduction and drug discontinuation are approximately 70% and 30%, respectively, due to the existence of cytopenia, renal dysfunction, rejection, or extrahepatic complications, and high levels of immunosuppression in this time window. The most recently published prospective, multicenter, randomized study (PHOENIX study) by Bzowej et al. [165] was designed to compare the efficacy, tolerability, and safety of an escalating dose regimen of PEG-INF alpha 2a/RBV for 48 weeks for preemptive antiviral treatment versus no treatment, which showed only 22% SVR in the prophylaxis patients with the rate of marked HCV recurrence at 120 weeks (62% in prophylaxis patients versus 65% in observation patients), and comparable fibrosis progression 120 weeks as well as similar patient/graft survival in both study arms. Dose reduction and discontinuation were required in 70% and 28%, respectively. Based on these results, European and United States transplant societies do not support the routine use of preemptive antiviral therapy.

Consequently, initiating antiviral therapy with PEG-INF/RBV after the confirmation of recurrent hepatitis C in the graft by liver biopsies is the mainstay for the treatment of recurrent disease in Western countries [35, 166–190]. Most of the data come from uncontrolled studies with different designs regarding time to start treatment, regimen used, and follow-up, but treatment duration is generally 48 to 52 weeks. Therefore, the results were also very different, with SVR rates ranging 0% to 56% (median: 33%), discontinuation rates ranging 4% to 58%, and dose reduction rate ranging 28% to 100%. In addition, the survival benefit of the treatment has not been confirmed in most studies so far, and it is compelling to conclude that there is currently no evidence to support the antiviral treatment for recurrent graft hepatitis C due to the lack of clinical benefit and frequent adverse effects, as concluded by the recent Cochrane meta-analysis [191]. On the other hand, recent retrospective cohort studies with a considerable follow-up duration found improved patient/graft survival in patients who obtained an SVR after antiviral treatment [35, 192–194]. Further randomized clinical trials with appropriate trial methodology and adequate follow-up duration are necessary to confirm an actual survival benefit of antiviral treatment.

## 6. Reports from Japanese LDLT Centers

Although retransplantation is the only potentially curative option for those with decompressed cirrhosis due to recurrent hepatitis C, in contrast to Western countries where re-DDLT is spared as a last resort [195, 196], it is extremely unlikely in Japan to perform retransplantation for patients with recurrent end-stage hepatitis C, if not absolutely impossible. These backgrounds might have led to various modified strategies for the treatment of recurrent disease as best efforts to secure first graft, such as aggressive preemptive antiviral treatment, escalation of dosages, and elongation of treatment duration.

We have reported preemptive INF/RBV treatment for HCV-positive LDLT recipients [161, 197–199]. Preemptive treatment was started just after recipient's condition had become stable (approximately one month after LDLT) with low-dose INF alpha 2b and RBV (400 mg/day) followed by escalation to PEG-INF (1.5 *μ*g/kg per week) and RBV (800 mg/day) depending on patient's tolerance. The treatment duration was not settled, and was continued for additional 12 months after the serum HCV-RNA became negative. The response was considered to be SVR provided negative serologic results for another 6 months after discontinuation of therapy. That is, nonstopping peg-INF/RBV approach was applied for non-responders. Among 122 HCV-positive LDLT recipients, 42 (34%) achieved SVR and those with SVR showed significantly improved survival when compared to those without SVR (cumulative 5-year survival rate; 97% versus 66%) [199].

Kyoto group also reported modified PEG-INF/RBV treatment with individualized extension, while they started antiviral treatment for cases with biopsy-proven recurrent disease [200–202]. They started with PEG-INF (1.5 *μ*g/kg per week) and RBV (400–800 mg/day) for 12 months for all patients with recurrent hepatitis. Then, full dose treatment was continued for additional 8–22 months for those whose serum HCV-RNA became negative within 12 months, while patients who did not become negative for serum HCV RNA within 12 months continued to receive a low-dose PEG-INF (0.5–0.75 *μ*g/kg per week) with or without reduced RBV (200 mg/day) as maintenance treatment. Among 80 patients with recurrent hepatitis C after LDLT, SVR was achieved in 31 (39%), while remaining 49 (61%) received maintenance therapy among those 26 (53%) discontinued. In comparison to fibrosis progression, no difference was observed between SVR group and maintenance treatment group with improved or stable fibrosis in both groups, while those who withdrew from maintenance showed significantly deteriorated fibrosis [202].

Kyushu group performed antiviral treatment for 80 patients among 106 consecutive HCV-positive recipients, excluding 26 cases of early death, negative HCV RNA, and refusal for treatment [203]. Basically, they started with PEG-INF (0.5 *μ*g/kg per week) and RBV (200 mg/day), then escalated to PEG-INF (1.5 *μ*g/kg per week) and RBV (800 mg/day), with the treatment duration of 48 weeks and over 72 weeks for those with early viral response and for those without it, respectively. They reported overall SVR rate of 35%. They found both significantly severe fibrosis and impaired graft survival in those who did not show viral nor biochemical response.

Other Japanese centers [204–207] have also reported similar modified antiviral treatment with PEG-INF and RBV including dose escalation, treatment for all HCV-positive cases, and extension of treatment. Additionally, simultaneous splenectomy during LDLT operation in an attempt to improve tolerance to antiviral treatment, SVR rate and further graft survival should be noticed [198, 208, 209].

## 7. Conclusion

Hepatitis C is here to stay and will remain the most common indication for liver transplantation. In the areas where cadaveric organs are extremely limited like in Japan, indication of LDLT is same as that of DDLT, and recent studies have proved that LDLT can be performed as safely and effectively as DDLT for HCV-infected patients in experienced centers. Further investigation for more effective and tolerable antiviral treatment is warranted to secure the first live donor graft to the possible extent.

## Figures and Tables

**Table 1:** Factors associated with the severity of recurrent hepatitis C after liver transplantation.

Variables	Effect on recurrent hepatitis C
Donor and graft factors	
Age [17, 37–43]	More severe disease (>40, >50, >65)
Steatosis [56, 57, 76–79]	Few studies
Prolonged ischemic time [54, 55, 80–83]	More severe disease
HCV+ graft [6, 22, 40, 50–53, 76]	No influence
Reduced size versus whole liver (LDLT versus DDLT) [39, 84–95]	No difference
Pretransplant recipient factors	
Genotype 1b [8, 32, 33, 35, 40]	Controversial
Pre-LT higher viral load [21, 28, 96, 97]	Unclear
Age [32, 44, 98]	Few studies
Race [45, 46, 99]	Few studies
Sex [20, 47, 48]	Few studies
HIV coinfection [100–107]	No influence
IL-28B gene polymorphism [49, 108–111]	More severe disease in CT and TT genotype
Posttransplant recipient factors	
Post-LT higher viral load [10, 27–31]	More severe disease
CMV infection [22, 29, 32, 112–116]	Unclear
Diabetes mellitus (Metabolic syndrome) [29, 117–121]	More severe disease
Immunosuppression	
Steroid bolus/OKT3 [6, 21, 22, 58, 59, 122–124]	More severe disease
Maintenance steroid [34, 60–62, 122]	Severe disease when rapidly tapered
Steroid free regimen [63–68, 125–127]	No influence
Tacrolimus versus cyclosporine [69–75]	No difference
Anti-IL-2 receptor antibodies [63, 126, 128–131]	Controversial
Azathioprine/mycophenolate mofetil [132–140]	Controversial
mTOR inhibitors [141–143]	Few studies

CMV: cytomegalovirus; DDLT: deceased-donor liver transplantation; HCV: hepatitis C virus; HIV: human immunodeficiency virus; LDLT: living-donor liver transplantation; LT: liver transplantation; mTOR: mammalian target of rapamycin.

**Table 2:** Studies comparing living-donor liver transplantation and deceased-donor liver transplantation in patients with hepatitis C cirrhosis.

Author	Year	*n* (LDLT/DDLT)	MELD score (LDLT/DDLT)	Donor age (LDLT/DDLT)	Cold ischemia time (h) (LDL/DDLT)	Follow-up (mo)	Histologic progression	Patient survival LDLT/DDLT (%)	Graft survival LDLT/DDLT (%)	Comments
Gaglio et al. [144]	2003	68 (23/45)	12.6/28*	NA	NA	24	NA	87/89	87/85	No difference in outcomes, increased risk of cholestatic hepatitis in LDLT
Garcia-Retortillo et al. [145]	2004	117 (22/95)	11 (5–24)/11 (2–28)	31 (19–58)/47 (13–86)^#^	NA	22	Significantly severe in LDLT	NA	NA	Severe hepatitis C recurrence in LDLT
Thuluvath and Yoo [146]	2004	619 (207/412)	NA	35.8 ± 0.4/38.9 ± 18.1^#^	3.9 ± 7.3/8.4 ± 4.5^†^	24	NA	79/81	74/73	Lower graft survival in LDLT
Humar et al. [85]	2005	51 (12/39)	17 (14–27)/24 (17–40)*	37.7 ± 9.2/42.8 ± 16.2^#^	10.2 ± 4.2/<1^†^	28.3	Significantly severe in DDLT	92/90	NA	LDLT may be at a low risk for HCV recurrence
Shiffman et al. [84]	2004	76 (23/53)	13.5 ± 1.1/16.2 ± 1.0	47.6 ± 2/47.8 ± 0.8	NA	36	No difference	79/82	76/82	No difference in outcomes
Maluf et al. [86]	2005	126 (29/97)	13.2 ± 1.1/21 ± 0.8*	NA	0.6 ± 0.2/7.5 ± 2.8^†^	72	NA	67/70	64/69	No difference in survival, more rejection in DDLT and biliary complications in LDLT
Russo et al. [87]	2004	4234 (279/3955)	NA (TB, PT and Cre were significantly worse in DDLT)	37/40^#^	8.1/2.6^†^	24	NA	83/81	72/75	No difference in outcomes
Bozorgzadeh et al. [88]	2004	100 (35/65)	14.9 ± 4/15.9 ± 5.3	34.6 ± 9.7/49.2 ± 20.4	NA	39	No difference	89/75	83/64	No difference in outcomes
Van Vlierberghe et al. [89]	2004	43 (17/26)	15 ± 9/15 ± 8	31 ± 8/48 ± 17	3.1 ± 1.3/11.1 ± 2.6^†^	12	No difference	No difference (Presented with only figure)	No difference (Presented with only figure)	No difference in outcomes in short-term
Schiano et al. [90]	2005	26 (11/15)	14 (9–19)/18 (10–31) *P* = 0.05	33 (20–54)/47 (13–73)	0.6 (0.3–1.0)/10 (4.4–20)^†^	24	NA	73/80	73/80	No difference in survival, accelerated viral load increase in LDLT
Guo et al. [91]	2006	67 (15/52)	16.9 ± 6.9/19.0 ± 8.3	NA	NA	24	No difference	93/96	87/94	No difference in outcomes
Terrault et al. [92]	2007	275 (181/94)	14 (6–40)/18 (7–40)*	38 (19–57)/41 (9–72)	0.8 (0.1–8)/6.7 (0.2–10)^†^	36	No difference	74/82	68/80	No significant difference in patient/graft survival in experienced LDLT centers
Schmeding et al. [93]	2007	289 (20/269)	NA	38.6 ± 15.2/44.2 ± 12	NA	60	No difference	Better in DDLT (*P* = 0.011)	Better in DDLT (*P* = 0.006)	LDLT does not increase the risk and severity of HCV recurrence. No difference in patient/graft survival when HCC beyond Milan excluded.
Selzner et al. [94]	2008	201 (46/155)	14 (7–39)/17 (6–40)	38 (19–59)/46 (11–79)^#^	1.5 (0.5–4.9)/7.5 (1.1–16)^†^	60	Significantly severe in DDLT	84/78	76/74	Donor age, rather than transplant approach, affects the progression of HCV
Gallegos-Orozco et al. [39]	2009	200 (32/168)	14.6 ± 4.7/25.5 ± 5.9*	35 ± 12/40 ± 16 *P* = 0.05	NA	60	No difference	81/81	NA	LDLT is a good option for HCV cirrhosis
Jain et al. [95]	2011	100 (35/65)	14.5 ± 3.9/16.8 ± 7.3*	34.3 ± 9.3/47.2 ± 19.8^#^	11 ± 3.1 in DDLT	84	Significantly severe in DDLT at all time points	77/65	71/46	Both patient/graft survival and histologic findings were better in LDLT

*MELD score is significantly higher in DDLT.

^#^Donor age is significantly higher in DDLT.

^†^Cold ischemia time is significantly longer in DDLT.

Cre: creatinine; DDLT: deceased-donor liver transplantation; LDLT: living-donor liver transplantation; MELD: model for end-stage liver disease; NA: not available; PT: prothombin-time; TB: total bilirubin.

## Abbreviations

DDLT: Deceased-donor liver transplantation
HCV: Hepatitis C virus
HIV: Human immunodeficiency virus
INF: Interferon
LDLT: Living-donor liver transplantation
MELD: Model for end-stage liver disease
MMF: Mycophenolate mofetil
mTOR: Mammalian target of rapamycin
PEG-INF: Pegylated-interferon
RBV: Ribavirin
RNA: Ribonucleic acid
SVR: Sustained viral response
UNOS/OPTN: The United Network for Organ Sharing/Organ Procurement and Transplantation Network.
